# Inertial Measurement Unit and Heart Rate Monitoring to Assess Cardiovascular Fitness of Manual Wheelchair Users during the Six-Minute Push Test

**DOI:** 10.3390/s24134172

**Published:** 2024-06-27

**Authors:** Grace Fasipe, Maja Goršič, Erika V. Zabre, Jacob R. Rammer

**Affiliations:** 1 Department of Biomedical Engineering, University of Wisconsin-Milwaukee, Milwaukee, WI 53211, USA; gorsic@uwm.edu (M.G.); ezabre@uwm.edu (E.V.Z.); jrrammer@uwm.edu (J.R.R.); 2 Department of Biomedical Engineering, Marquette University, Milwaukee, WI 53233, USA

**Keywords:** inertial sensor, heart rate, manual wheelchair, six-minute push test, inertial measurement unit, mobility, physical fitness

## Abstract

Manual wheelchair users (MWUs) are prone to a sedentary life that can negatively affect their physical and cardiovascular health, making regular assessment important to identify appropriate interventions and lifestyle modifications. One mean of assessing MWUs’ physical health is the 6 min push test (6MPT), where the user propels themselves as far as they can in six minutes. However, reliance on observer input introduces subjectivity, while limited quantitative data inhibit comprehensive assessment. Incorporating sensors into the 6MPT can address these limitations. Here, ten MWUs performed the 6MPT with additional sensors: two inertial measurement units (IMUs)—one on the wheelchair and one on the wrist together with a heart rate wristwatch. The conventional measurements of distance and laps were recorded by the observer, and the IMU data were used to calculate laps, distance, speed, and cadence. The results demonstrated that the IMU can provide the metrics of the traditional 6MPT with strong significant correlations between calculated laps and observer lap counts (r = 0.947, *p* < 0.001) and distances (r = 0.970, *p* < 0.001). Moreover, heart rate during the final minute was significantly correlated with calculated distance (r = 0.762, *p* = 0.017). Enhanced 6MPT assessment can provide objective, quantitative, and comprehensive data for clinicians to effectively inform interventions in rehabilitation.

## 1. Introduction

Wheelchair propulsion is a primary and crucial mode of ambulation for many individuals with mobility impairments. With wheelchair users’ more sedentary lifestyle and limited mobility [1], high levels of functional maneuverability and cardiovascular health are pivotal for their quality of life and general well-being [2]. There are an estimated 3.3 million wheelchair users in the United States [3] who could benefit from improved cardiovascular fitness assessments. Such an assessment could identify levels of fitness and movement capabilities, providing clinicians with appropriate tools to prescribe and promote regular physical activity, decreasing the risk of heart disease and enhancing mobility and functional independence [4,5].

One mean of measuring both the physical fitness and functional independence of manual wheelchair users (MWUs) is the six-minute push test (6MPT) [6,7], adapted for MWUs based on the six-minute walk test (6MWT), a standard outcome assessment used for ambulatory individuals [8]. During the 6MPT, the wheelchair user propels themselves as far and as fast as they can in 6 min. Typically, the test is conducted in the clinic and requires the MWU to perform as many fixed distance laps as they are able to in the available time [7]. Generally, recorded distances above 604 m and 445 m indicate good fitness levels for paraplegic and tetraplegic individuals, respectively [9]. Heart rate (HR) of the MWU is recorded prior to the test to estimate their resting heart rate and after the test as a measure of physical exertion. It is used alongside the speed to calculate the maximal oxygen consumption (VO_2max_), allowing clinicians and researchers to evaluate MWUs’ endurance, heart health, and heart rate [6,7,10]. The 6MPT is highly reliable [11] and has been shown to be sensitive in determining cardiovascular fitness in individuals with spinal cord injuries [9]. Both the 6MWT and 6MPT have been used to establish a baseline for clinicians to best assign rehabilitation and training programs for their patients while measuring the progress and effectiveness of the interventions prescribed to their patients [6,7,8,10].

Although the 6MPT and 6MWT are simple and easy to conduct, they have several limitations, which can noticeably affect the effectiveness of the assessment. As the tests are traditionally conducted by the clinician for measuring timing and distance traveled during the test, observer bias and a certain level of imprecision for the total distance traveled is present [9]. For the 6MPT, the observer-reported data can be considered subjective due to variability in interpretation and experience level. Physical activity assessment of MWUs is particularly challenging due to the differences in propulsion patterns, emphasizing the need for accurate measurement tools [12]. To address these limitations, accelerometers have been introduced in conjunction with the 6MWT to enable more accurate data collection by directly measuring the distance walked [13,14,15,16]. The sensor can provide detailed insights and measurements of step length and overall distance traveled during the 6MWT, highlighting the feasibility of using the sensor to quantify physical activity.

Accelerometers have also been widely used to capture objective data from MWUs. They have been leveraged to analyze wheelchair propulsion kinematics and kinetics, offering insights into the physical demands placed on MWUs during daily activities and exercise [17,18,19]. Additionally, research demonstrated the use of accelerometers to measure speed, acceleration, and push frequency of MWUs [20,21], all of which are considered critical factors for evaluating the efficiency and safety of wheelchair propulsion. Through the attachment of accelerometers to the wheelchair on the wheels or seat, or to the MWUs’ upper arms, forearms, and chests, previous studies have been able to utilize the accelerometer data to identify movement patterns and potential strain on the musculoskeletal system, enabling personalized interventions to prevent injury and enhance performance outcomes [20,21,22]. Furthermore, inertial measurement units (IMUs), which integrate accelerometers, gyroscopes, and magnetometers, have been used to provide real-time feedback to wheelchair users, aiming to optimize their propulsion technique and reduce the risk of overuse injuries, particularly in the shoulder [20,22,23,24]. 

One of the key metrics utilized in load monitoring for MWUs is heart rate [25]. Heart rate monitors are commonly used in measuring training intensity [26,27], general exercise intensity [28], as well as to provide insights into the perceived exertion of the MWU [29,30]. Heart rate-based formulas can estimate the VO_2_ of a wheelchair user, though effectiveness of this method can be impacted by the type of MWU condition [31]. They have also been utilized during the 6MPT assessment for MWUs and during 6MWT assessments, though heart rate monitors have been shown to have errors in registering heart rate [7,32,33,34,35].

Despite the use of the inertial sensor in the 6MWT and the availability of the IMU sensor in wheelchair studies, their potential to provide objective data during the 6MPT has not yet been explored. The objective data collected by the IMUs are the quantifiable measurement through the IMU’s sensors, which provide an unbiased representation of physical activity rather than relying upon an observer’s subjective perspective. While the 6MPT traditionally measures distance and is often paired with a heart rate monitor, inertial sensors offer the currently untapped potential to capture detailed objective spatial and temporal data, that, alongside the heart rate data, could enhance the understanding of wheelchair propulsion and user-specific functional abilities, which this study hopes to elucidate.

This study will explore the viability and effectiveness of utilizing IMU sensors in quantifying variables during the 6MPT, a standardized assessment used for evaluating MWU’s physical and functional health. Utilizing objective measurements from IMUs, as well as the cardiovascular data from the heart rate monitor, the 6MPT has the potential to provide a more comprehensive assessment to clinicians regarding the physical fitness, functional independence, and cardiovascular health of their MWU patients [36,37,38].

## 2. Materials and Methods

### 2.1. Participants

Ten adult MWU participants (7 male, 3 female, aged 34 ± 9 years, BMI 28.8 ± 7.1 kg/m^2^) who reported no history of shoulder injury within the past year were recruited for this study. All participants were required to regularly use a manual wheelchair for their daily mobility needs. This study was approved by the University of Wisconsin-Milwaukee Institutional Review Board (protocol #24.059). Participants met with the research team where they had the study protocol explained and signed a written informed consent document prior to participation. The demographics of all participants are presented in Table 1, including their gender, age, BMI, injury type, and their wheelchair propulsion pattern.

### 2.2. Hardware

Two commercially available triaxial Blue Trident IMUs (Vicon Motion Systems Ltd., Oxford, UK) were utilized during this study to record linear acceleration and angular velocity of the participant. The IMU has the accelerometer and gyroscope sensors integrated with the same coordinate axes. The recorded accelerometer and gyroscope data were stored on the IMU and later downloaded for further analysis by connecting the IMU to the provided Capture.U phone app through Bluetooth. A commercially available Garmin Forerunner 735XT (Garmin Ltd., Olathe, KS, USA) wristwatch was used to track participants’ heart rates. The heart rate data were downloaded from the Garmin Connect phone app via Bluetooth to a personal computer for data analysis.

### 2.3. Study Protocol

#### 2.3.1. IMU and Heart Rate Monitor Placement

**Figure 1:**
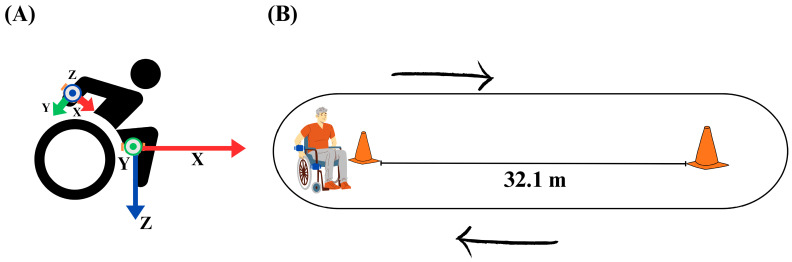
Schematic representation of 6MPT layout. (**A**) Vector direction of the IMU on the wrist and wheelchair of the participant, indicating the axes of both the accelerometer and gyroscope. (**B**) Outline of experimental setup, where two traffic cones were placed 32.1 m apart and the participant wheeled between and around them.

Based on the objectives of this study, two IMUs were chosen to measure specific and complementary aspects of the 6MPT and the MWU’s mobility metrics. For each participant, one IMU was placed on the bottom of the participant’s personal wheelchair and oriented with the *x*-axis pointing forward, parallel with the wheelchair’s forward direction of motion, and the *z*-axis pointing down parallel with the direction of gravity. This IMU was positioned to provide consistently oriented data on the forward movement of the wheelchair as well as the turning angular velocity during the trial. Studies have used wheelchair-mounted IMUs to derive mobility metrics applicable to our study’s goals [18,39]. The second IMU was mounted on the participant’s right wrist using a wrist strap and oriented with the *x*-axis pointing in the same direction as the participant’s thumb and the *z*-axis pointing outwards from the participant’s forearm. The wrist was chosen due to its proximity to the rim of the wheelchair and because it provides a direct measure of the arm movement, such as cadence, during the trial. Research has shown that IMUs placed on the wrist are more effective at measuring repetitive arm motion, a crucial element of manual wheelchair propulsion [40]. Figure 1A shows the positioning of the IMU on the participant and the wheelchair. Previous research into the measurement of accelerometer data for wheelchair users indicates that there is little to no difference between the dominant and non-dominant wrist [41]. The Garmin wristwatch was also strapped to the right wrist of the participant. Both the wristwatch and the IMU were secured to ensure that they would not come loose during the trial or collide with each other during wheelchair propulsion. The IMU and heart rate monitor placement were consistent for all participants to maintain consistency in the measurements during the trial.

#### 2.3.2. Six-Minute Push Test

Since the 6MPT has been modeled after the 6MWT [7], American Thoracic Society guidelines and instructions [42] were followed for its administration. The 6MPT is a standard clinical protocol that is widely recognized in assessing physical and cardiovascular fitness for MWUs. It is typically performed as a single 6-min trial per participant and considered sufficient for assessing physical fitness. In a long hallway with a smooth, tiled surface, two cones were positioned 32.1 m apart from each other. The participant was positioned next to one cone facing the second cone. The participant was instructed to propel themselves as rapidly as they could to the second cone, circle it, and return to the first, repeating the process as many times as they can within the trial’s 6-min time frame (Figure 1B). During the 6MPT, the observer counted the laps and verbally notified the participant at the 3 min, 5 min, and 5 min 30 s marks of the trial. At the 6-min mark, the observer instructed the participant to come to a complete stop before the sensors were removed from both the participant’s wrist and the wheelchair. Heart rate was continuously measured for 5 min before the trial as well as during the entirety of the 6MPT. To ensure consistency and minimize bias, we aimed to keep the test conditions as similar as possible across all participants following the clinical standard of the test. This includes consistent instruction and uniform verbal queues to minimize variability in the test administration.

### 2.4. Data Analysis

The primary outcome metrics calculated from the IMUs during the 6MPT were the participant’s speed, cadence, number of laps, and total distance traveled. The heart rate monitor was used to obtain the overall mean and maximum heart rates and mean heart rate during the final minute of the 6MPT. The heart rate was used to estimate the VO_2max_ during the trial. The observed metrics (i.e., from the observer) were the counted laps and the resulting calculated distance traveled.

Data processing was performed using custom algorithms in Matlab software version R2023b (MathWorks Inc., Natick, MA, USA). The beginning of the trial was determined by the observer giving a verbal command to begin, upon which the participant started moving. For each IMU, the data were analyzed to detect a change in acceleration, which determined the start of the trial. The wrist IMU accelerometer data were utilized to calculate the cadence of the participant, defined as the strokes of the user’s hand in contact with the wheel per minute. The wheelchair IMU’s accelerometer and gyroscope data were used to calculate the number of laps, speed, and distance during the trial. The IMU and heart rate data were analyzed separately and manually synchronized in the same manner for each participant.

To reduce noise, accelerometer and gyroscope data were filtered with a fourth-order, low-pass Butterworth filter (6 Hz). In order to remove the inherent bias and effect of gravity on the IMU data, the mean was subtracted from the data. The following sections detail the specifics as to how the different parameters were calculated.

#### 2.4.1. Number of Laps

**Figure 2:**
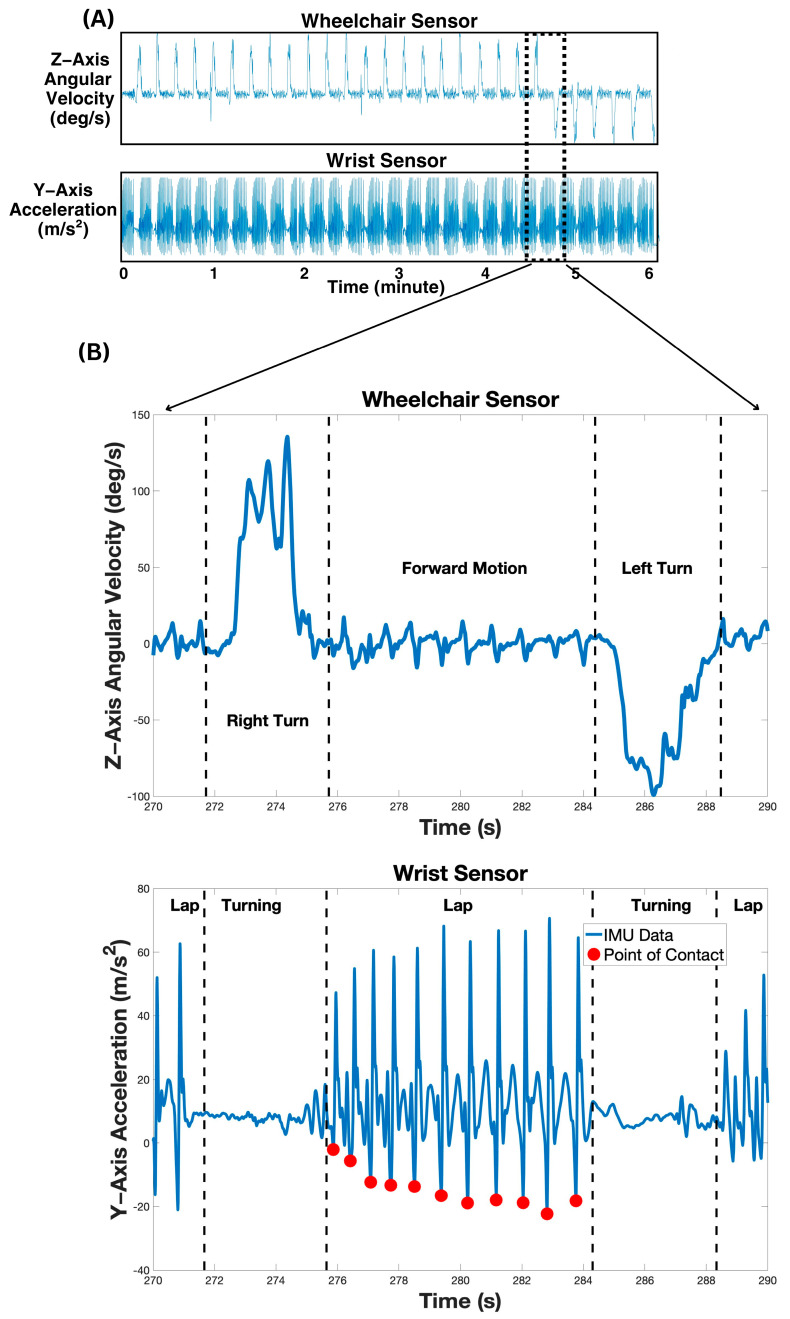
IMU data processing. (**A**) Overall 6MPT *z*-axis angular velocity (deg/s) and *y*-axis acceleration (m/s^2^) data. (**B**) One lap sample for identification of turns and point of contact (red dots).

The *x*-axis acceleration from the wheelchair IMU was utilized to identify the beginning timestamp of the 6MPT in the recorded data, marked by a sudden and sharp increase in *x*-axis acceleration compared to the resting acceleration prior to the start of the 6MPT. This increase was identified using “ischange” Matlab function. The *z*-axis angular velocity from the wheelchair IMU was plotted from the start of the trial to the end of the 6 min (see Figure 2A). From the plotted *z*-axis data, the number of laps completed by the participant was identified by sudden and sharp increases in *z*-axis angular velocity, indicating a turn around one of the two cones (see Figure 2B). Recognition of the turnaround points was conducted using “local maxima” function in Matlab with the minimum peak set to 60% of the maximum angular velocity of the trial. Furthermore, the minimum interval for the local maxima function was set to 7.2 s based on a reasonable estimate of how many laps could feasibly be completed in 6 min and Olympic records for the fastest speeds recorded by an MWU [43].

#### 2.4.2. Distance

The calculated distance from the IMU data was obtained by multiplying the total calculated laps by the known distance of 32.1 m between the two cones. To account for the final partial lap, a piecewise double integration of the acceleration in the *x*-axis of the wheelchair IMU was conducted between the final detected turn and the end at 6 min. This method was only used during the final partial lap to minimize error accumulation from drift, a common issue encountered with continuous integration of IMU data.

#### 2.4.3. Speed

For both the observed and IMU data, the average speed for each participant was calculated by dividing the distance traveled by the 6 min, while the average lap speed as measured by the IMU was calculated by dividing the lap distance by the lap time.

#### 2.4.4. Propulsion Cycle and Cadence

To identify the contact and recovery phases of an MWU’s propulsion cycle, a steady-state propulsion cycle was recorded. The propulsion cycle was isolated from the wrist IMU accelerometer data and synchronized with a recorded video. The two phases of the propulsion cycle, namely the contact phase and recovery phase, were visually identified from the video recording when the hand made contact with the push rim of the wheel and when it left the push rim of the wheel, respectively. This was utilized in conjunction with the plotted *y*-axis acceleration data from the wrist IMU (see Figure 2A) to isolate the typical peaks and troughs associated with the contact and recovery phases. The “local maxima” function in Matlab with a minimum separation set to half a second and a minimum peak of a quarter of the highest recorded angular velocity in the *y*-axis was used for phase detection of the trial data. This phase detection was utilized for each participant to automatically recognize and count each propulsion cycle (see Figure 2B). Each participant’s cadence (cycles/minute) was calculated by dividing the total detected propulsion cycles by the 6 min duration of the trial.

#### 2.4.5. Heart Rate

The overall mean, minimum, and maximum heart rates for the whole 6MPT as well as the mean heart rate during the final minute of the test were calculated based on the recorded heart rate data for each participant from the Garmin wrist photoplethysmography (PPG) sensor. An estimate of the VO_2max_ was obtained by applying the Heart Rate Ratio Method [44,45] to the resting heart rate and the max heart for the whole trial (Equation (1)).

(1) $$VO_{2\max}=\frac{HR_{\max}}{HR_{\mathrm{rest}}}\cdot 15\;\frac{\mathrm{mL}}{\mathrm{kg}\;\min}$$

#### 2.4.6. Conventional Measurements

The participant’s distance from the observer’s counted laps was calculated by multiplying the total laps by the distance between the cones (32.1 m). If the participant was observed to complete at least half of a lap during the final partial lap, the observer noted it as 0.5 for the final count.

### 2.5. Statistical Analyses

All statistical analyses were performed using SPSS software (version 28, SPSS Inc., Chicago, IL, USA). The descriptive statistics were calculated across all participants. The measurement variables were assessed for normality using the Shapiro–Wilk test. To identify any correlations between the IMU data and the conventional 6MPT data, Pearson correlation analysis was performed between the IMU and observer laps and distances. Furthermore, Pearson correlations were calculated among the IMU laps and distances and the heart rate data to explore further relationships. Correlation coefficients (r) greater than 0.7 indicated a strong correlation, following existing guidelines and related research [11,46,47,48,49], and a *p*-value less than 0.05 indicated significance. The sample size varied between *n* = 9 and *n* = 10 for different correlations due to missing heart rate data for Participant 5.

## 3. Results

### 3.1. Six-Minute Push Test Data

The data observed during the 6MPT and the metrics calculated from the IMU and from the wristwatch are presented in Table 2 for all 10 participants during the 6MPT. The wristwatch failed to collect the heart rate for Participant 5. 

Figure 2 displays an example of a typical trial’s IMU data for one participant and summarizes the steps for analyzing the *z*-axis gyroscope from the wheelchair IMU for identifying the turnaround points and marking the beginning of each new lap. It also displays the data from the *y*-axis accelerometer of the wrist IMU and summarizes the steps for analyzing the data to identify each propulsion cycle with each contact phase for one lap (marked by red dots).

**Figure 3:**
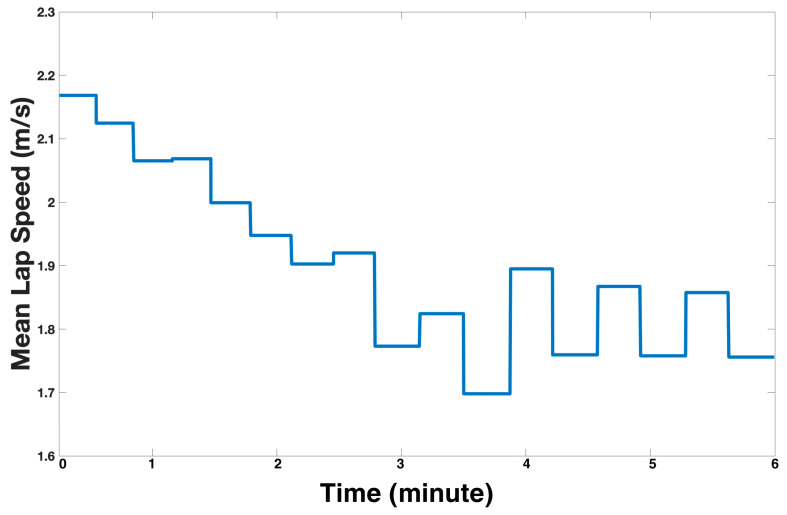
Individual lap IMU mean speed for one participant during the 6MPT.

The mean speed of each lap from the IMU on the wheelchair of one participant is displayed in Figure 3 where each step represents a completed lap, illustrating the participant’s fluctuating speed throughout the course of the 6MPT, with a noticeable decreasing trend.

**Figure 4:**
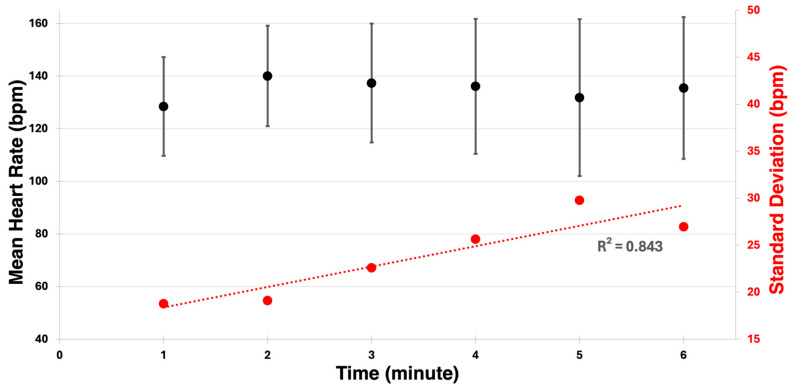
Mean and standard deviation of heart rate across all participants. A positive linear fit of the standard deviation is shown.

The heart rates averaged across participants for each minute are shown in Figure 4, illustrating an increase in variability throughout the trial. The standard deviation of heart rate showed a consistent increase over time, supported by a strong linear fit with an R-squared value of 0.843.

### 3.2. Statistical Results

Table 3 lists the statistical results of the Pearson correlation analysis. The missing heart rate data for Participant 5 did not affect the overall pattern of significant correlations observed in the other metrics. The IMU calculated laps had a strong positive correlation with the observer lap counts (r = 0.947, *p* < 0.001) and distances (r = 0.970, *p* < 0.001). Heart rate during the final minute of the 6MPT also exhibited strong positive correlations with the IMU calculated distance (r = 0.762, *p* = 0.017) and laps (r = 0.756, *p* = 0.018). However, neither the IMU laps nor distance showed a statistically significant correlation with either the overall mean or maximum heart rate, though they were moderately correlated (for laps: mean HR—r = 0.513, *p* = 0.158, max HR—r = 0.407, *p* = 0.276, and for distance: mean HR—r = 0.543, *p* = 0.131, max HR—r = 0.437, *p* = 0.239). The mean speed was also found to be strongly correlated with the mean heart rate during the final minute of the trial (r = 0.758, *p* = 0.018). The VO_2max_ did not have significant correlations with any variables (0.103 > r > 0.619, 0.075 < *p* < 0.792).

## 4. Discussion

The main objective of this study was to explore the viability of integrating the IMU sensor into the 6MPT, demonstrating its effectiveness in tracking objective, quantitative data traditionally recorded manually by an observer during a conventional 6MPT. Also, the study aimed to examine potential correlations between the IMU output and the heart rate measurements to provide more insights into the cardiovascular fitness and exertion of the participants. The strong correlations found between the IMU calculated laps and the observer lap counts (r = 0.964, *p* < 0.001) and distances (r = 0.971, *p* < 0.001) suggest that it is feasible for the IMU sensors to provide objective, quantitative results for lap measurement, distance assessment, and average speed during the 6MPT (see Table 3). Furthermore, during the trials, there were instances where the observer counted laps deviated from the IMU calculated laps. This is an example of how observer bias and error can impact the results of the 6MPT, whereas the IMU may provide more objective data and reduce the potential for user errors [50].

Utilizing the IMU during the 6MPT has the potential to ease the testing process and provide objective, quantitative data. This study’s findings demonstrated that the IMU can measure critical physical fitness-related metrics, which would not have otherwise been obtained during the 6MPT, such as cadence and vector magnitude. Previous research has identified cadence as one of the risk factors for overuse and shoulder injury in MWUs [18,51]. The IMU provides the means to calculate the cadence of the MWU. The cadence provides an objective measure of MWU propulsion, which is related to propulsion patterns and arm kinematics. Furthermore, cadence can be used to improve MWU propulsion, minimize the risk of overuse and upper extremity muscle demands, and optimize their rehabilitation goals [1,20]. While it is possible to obtain the cadence during the 6MPT without any instrumentation, the manual approach can be tedious and highly inaccurate due to the likelihood of observer error. The use of the IMU automates the cadence count, which could be used in the analysis and improvement of participant propulsion patterns [17,19]. 

Similarly, the IMU data allowed for the recording of the mean speed for each individual lap (see Figure 3), providing insight into how the participant’s performance fluctuates over the 6 min and potentially vital information into their physical fitness and movement biomechanics. In this case, the participant’s average lap speed clearly decreased through the trial, likely indicating the onset of fatigue as the trial progressed. Another aspect of MWU physical fitness that would otherwise be missed during a traditional 6MPT is the detailed movement of the participant’s wrist. With the IMU triaxial acceleration data, the vector magnitude can be obtained and has been shown to be useful in assessing movement intensity-related physical fitness and capabilities [14,23].

The relationship between the IMU data and heart rate metrics was explored next with significant correlations observed among several parameters. The heart rate during the final minute of the 6MPT was significant and highly correlated when compared with the IMU calculated distance (r = 0.762, *p* = 0.017), whereas the IMU-measured physical activity parameters were not significantly predictive indicators of overall mean or maximum heart rates (0.407 > r > 0.543, 0.131 < *p* < 0.276). These contrasting relationships have been previously established when utilizing the 6MWT [33,34,35], and these results point to the same relationships holding true for MWUs and the 6MPT. The change in statistical correlations may be due to the participant being made aware that the trial was nearly complete; as Figure 4 shows, the variation in heart rate increased as the trial progressed. Of note is the fact that the estimated VO_2max_ variable lacked a statistically significant correlation with any other measured variable, possibly due to the estimation lacking the accuracy of direct VO_2max_ measurement and not being tuned for MWUs. The inclusion of a direct method of measuring VO_2max_ during the 6MPT should be considered for future studies.

While these results are encouraging, it is critical to recognize the limitations of the study. Future studies will need to include a larger sample of participants, including wheelchair users with different levels of mobility and across different ages (pediatric, elderly) to ensure the results apply to the larger population. Furthermore, while it is important to acknowledge that IMUs can have accuracy and precision deficiencies [18,19,20,23,52], the impact of this on the study’s results is negligible due to it only examining spatiotemporal parameters. Additionally, combining accelerometer and gyroscope sensors with the 6MPT does increase cost and complexity of the testing process. This could be mitigated through the utilization of everyday technologies such as smartphones. Most smartphones have already integrated accelerometer and gyroscope sensors, and many modern smartwatches are capable of tracking heart rate. Future work could implement mobile phone applications for measuring the acceleration and angular velocity of the participant during the 6MPT. This would broaden usability of the test while enabling remote assessment of 6MPT metrics outside the clinical environment. Furthermore, future studies could include a physiological scale, such as the Borg or OMNI Exertion scales, to account for metrics outside of the capabilities of an IMU to provide a more holistic view of the participants’ physical states. 

The practical implications of our findings suggest that IMUs could be a valuable tool for clinicians and researchers who require objective, reliable, and efficient ways to quantitatively measure physical activity and cardiovascular fitness for MWUs. Combining IMU and heart rate sensors with the 6MPT aligns with precision medicine and personalized health, where individual data could be used for tailored interventions and to track progress over time [53,54,55]. In rehabilitation contexts, especially where the 6MPT is used to assess functional mobility, the utilization of IMU sensors could streamline the assessment process, allowing for real-time data analysis and immediate feedback to patients [12,56]. Furthermore, proper utilization of the IMU’s capability to measure the cadence and acceleration vector magnitude of the participant during the trial has the potential to allow rehabilitation specialists to be able to more effectively assess their patient’s propulsion pattern and guide them towards more effective and safer movement capability.

## 5. Conclusions

Our study provided evidence-based support for the use of IMU sensors to complement traditional observer-based methods in measuring MWU performance during a 6MPT. By measuring distances, speeds, and cadences, IMUs add important metrics for assessing the functional mobility of wheelchair users. One of the pivotal considerations in using IMUs is in ensuring their effectiveness compared to established methods. The strong correlations observed in our study provided evidence supporting the effectiveness of IMUs. This was consistent with previous research, which suggests that IMUs are effective tools for motion analysis in sports and rehabilitation settings [13,14,15,16,57]. However, our study also highlighted the need for multi-modal data collection strategies to account for all physiological aspects of the test. Overall, the use of IMU sensors during the 6MPT protocol offers a step forward in MWU rehabilitation assessment. This method not only aligns with the increased use of telehealth interventions but also empowers clinicians and users by providing detailed, objective, and actionable data, paving the way for personalized and adaptive rehabilitation strategies. 

## Figures and Tables

**Table 1:** Participant demographics. SCI—spinal cord injury, BMI—body mass index, C—cervical, T—thoracic, and L—lumbar.

ID	Gender	Age	BMI	Injury Type	Propulsion Pattern
1	Male	41	23.6	L3 Incomplete Injury	Semicircular
2	Male	26	32.4	T10 Incomplete Injury	Semicircular
3	Male	32	40.8	Sacral Spina Bifida, Incomplete SCI	Arc
4	Male	39	29.7	T8 Complete Injury	Arc
5	Male	38	19.4	T6 Complete Injury	Arc
6	Female	22	23.3	Neuromuscular Autoimmune Injury	Semicircular
7	Female	22	39.5	Spina Bifida L3, 4, 5	Semicircular
8	Male	47	28.2	C6/C7 Incomplete	Arc
9	Female	43	28.3	Spina Bifida T12	Semicircular
10	Male	30	22.9	Spinal Cord T10–12 Incomplete	Semicircular

**Table 2:** Six-minute push test observer and sensor variables for all 10 participants. HR—Heart rate, FMM—final minute mean.

ID#	Observer Data		IMU Data				Watch Data			
	Counted Laps	Distance (m)	Calculated Completed Laps	Distance with Partial Lap (m)	Mean Speed (m/s)	Cadence (cycles/min)	Mean HR (bpm)	Max HR (bpm)	FMM HR (bpm)	VO_2max_
1	32	1027.2	29	955.2	2.65	50.5	150.5	179	170.9	28.2
2	27	866.7	26	842.6	2.34	51.2	130.0	152	134.5	27.1
3	28	898.8	28	909.3	2.53	63.8	118.3	142	125.2	25.7
4	25	802.5	24	777.3	2.16	80.3	136	172	152.9	27.9
5	23.5	754.4	23	743.7	2.07	56.0	-	-	-	-
6	20.5	658.1	20	643.9	1.79	61.0	99.2	112	106.3	23.3
7	22.5	722.3	22	710.3	1.97	53.5	127.2	159	146.7	26.9
8	18	577.8	17	556.3	1.55	32.3	98.6	152	84.1	29.9
9	29	930.9	29	931.0	2.59	58.8	102.6	154	141.1	30.2
10	25.5	818.6	27	879.9	2.44	69.5	127.1	159	157.9	31.6
$\bar{X}$ (σ)	25.1 (4.2)	805.8 (133.6)	25 (4)	794.0 (130.8)	2.21 (0.36)	57.7 (12.7)	121.1 (17.9)	153 (19)	135.5 (27.0)	27.9 (2.5)

**Table 3:** Pearson correlation coefficients (r) between study variables. HR—Heart rate, IMU—inertial measurement unit.

Measure	Observer Laps	IMU Laps	Observer Distance (m)	IMU Distance (m)	Mean Speed (m/s)	Mean HR (bpm)	Max HR (bpm)	Final Mean HR (bpm)	VO_2max_
Observer Laps	1								
IMU Laps	0.947 **	1							
Observer Distance (m)	1.000 **	0.947 **	1						
IMU Distance (m)	0.969 **	0.997 **	0.970 **	1					
Mean Speed (m/s)	0.970 **	0.997 **	0.970 **	1.000 **	1				
Mean HR (bpm)	0.613	0.513	0.614	0.543	0.539	1			
Max HR (bpm)	0.493	0.407	0.494	0.437	0.435	0.746 *	1		
Final Mean HR (bpm)	0.754 *	0.756 *	0.754 *	0.762 *	0.758 *	0.831 **	0.685 *	1	
VO_2max_	0.162	0.276	0.162	0.258	0.257	0.103	0.619	0.277	1

Notes: ** Significance at the 0.01 level (two-tailed). * Significance at the 0.05 level (two-tailed).

## Data Availability

The data associated with this study are available upon reasonable request from the corresponding author.
